# Serum Occludin Level Combined With NIHSS Score Predicts Hemorrhage Transformation in Ischemic Stroke Patients With Reperfusion

**DOI:** 10.3389/fncel.2021.714171

**Published:** 2021-08-12

**Authors:** Shuhua Yuan, Weili Li, Chengbei Hou, Huining Kang, Qingfeng Ma, Xunming Ji, Zhifeng Qi, Ke Jian Liu

**Affiliations:** ^1^Cerebrovascular Diseases Research Institute, Xuanwu Hospital of Capital Medical University, Beijing, China; ^2^Laboratory of Brain Disorders, Ministry of Science and Technology, Collaborative Innovation Center for Brain Disorders, Beijing Institute of Brain Disorders, Capital Medical University, Beijing, China; ^3^Center for Evidence-Based Medicine, Xuanwu Hospital, Capital Medical University, Beijing, China; ^4^Department of Internal Medicine, University of New Mexico, Albuquerque, NM, United States; ^5^Department of Neurology, Xuanwu Hospital of Capital Medical University, Beijing, China; ^6^Department of Pharmaceutical Sciences, College of Pharmacy, University of New Mexico Health Sciences Center, Albuquerque, NM, United States

**Keywords:** occludin, hemorrhagic transformation, acute ischemic stroke, blood-brain barrier, NIHSS score

## Abstract

Hemorrhagic transformation (HT) is a severe complication following acute ischemic stroke, particularly with reperfusion interventions, leading to poor prognosis. Serum occludin level is related with blood brain barrier disruption, and the National Institute of Health stroke scale (NIHSS) score reflects stroke severity. We investigated whether the two covariates are independently associated with HT and their combination can improve the accuracy of HT prediction in ischemic stroke patients with reperfusion therapy. Seventy-six patients were screened from the established database of acute ischemic stroke in our previous study, which contains all clinical information, including serum occludin levels, baseline NIHSS score, and hemorrhagic events. Multivariate logistic regression analysis showed that serum occludin level (OR = 4.969, 95% CI: 2.069–11.935, *p* < 0.001) and baseline NIHSS score (OR = 1.293, 95% CI 1.079–1.550, *p* = 0.005) were independent risk factors of HT after adjusting for potential confounders. Compared with non-HT patients, HT patients had higher baseline NIHSS score [12 (10.5–18.0) versus 6 (4–12), *p* = 0.003] and serum occludin level (5.47 ± 1.25 versus 3.81 ± 1.19, *p* < 0.001). Moreover, receiver operating characteristic curve based on leave-one-out cross-validation showed that the combination of serum occludin level and NIHSS score significantly improved the accuracy of predicting HT (0.919, 95% CI 0.857–0.982, *p* < 0.001). These findings suggest that the combination of two methods may provide a better tool for HT prediction in acute ischemic stroke patients with reperfusion therapy.

## Introduction

Ischemic stroke accounts for 70–80% of all stroke patients, and reperfusion therapy has proved to be an effective treatment in patients with ischemic stroke (Wang et al., 2017; Campbell et al., 2019). Hemorrhagic transformation (HT) is one of the common complications of acute ischemic stroke, particularly after reperfusion therapy, leading to poor prognosis. Reperfusion therapy, such as mechanical thrombectomy or intravenous thrombolysis, increases the risk of HT, which is a major reason for withholding the beneficial therapy from most stroke patients (Yaghi et al., 2015). Therefore, it is critical to investigate how to predict HT in patients with acute ischemic stroke before reperfusion therapy.

Breakdown of the blood–brain barrier (BBB) is associated with the risk of HT in acute ischemic stroke (Kazmierski et al., 2012). As a member of tight junction proteins, occludin plays an important role in keeping BBB integrity (Hawkins and Davis, 2005). Our recent study with a rat model of ischemic stroke showed that occludin degradation occurred in the early phase of ischemic stroke and contributed to BBB disruption (Pan et al., 2017), suggesting that occludin might be a potential biomarker for predicting the risk of HT. Our clinical study reported that the serum occludin level, being significantly higher in patients with HT than those without HT, could reasonably predict HT in patients with reperfusion therapy (Li et al., 2020). We hypothesize that the combination of serum occludin level with other HT risk factors may further improve the predictive ability for HT in stroke patients with reperfusion therapy.

As it is well recognized, besides different treatment measures, there are many other risk factors associated with HT in clinic, such as age, ischemic severity, infarct size, hyperglycemia, and hypertension (Whiteley et al., 2012). Among them, neurological dysfunctions (as represented by the National Institutes of Health Stroke Scale, NIHSS score) and infarct size were most associated with HT (Emberson et al., 2014; Tan et al., 2014), and NIHSS score is known to be a good and stable predictor for bleeding transformation.

Therefore, the present study aims to determine whether serum occludin level combined with NIHSS score can improve the predictive value of HT in stroke patients with reperfusion therapy, utilizing our previous study database. The outcome of this study will be helpful for selecting the maximum number of patients who can benefit from reperfusion therapy while minimizing the risk of HT at the same time.

## Materials and Methods

### Data Source and Case Enrollment

The data in this study were obtained from our established database from a single-institute retrospective study (Li et al., 2020), in which all patients were from the Emergency Department at Xuanwu Hospital of Capital Medical University between November 2018 and March 2019.

**FIGURE 1:**
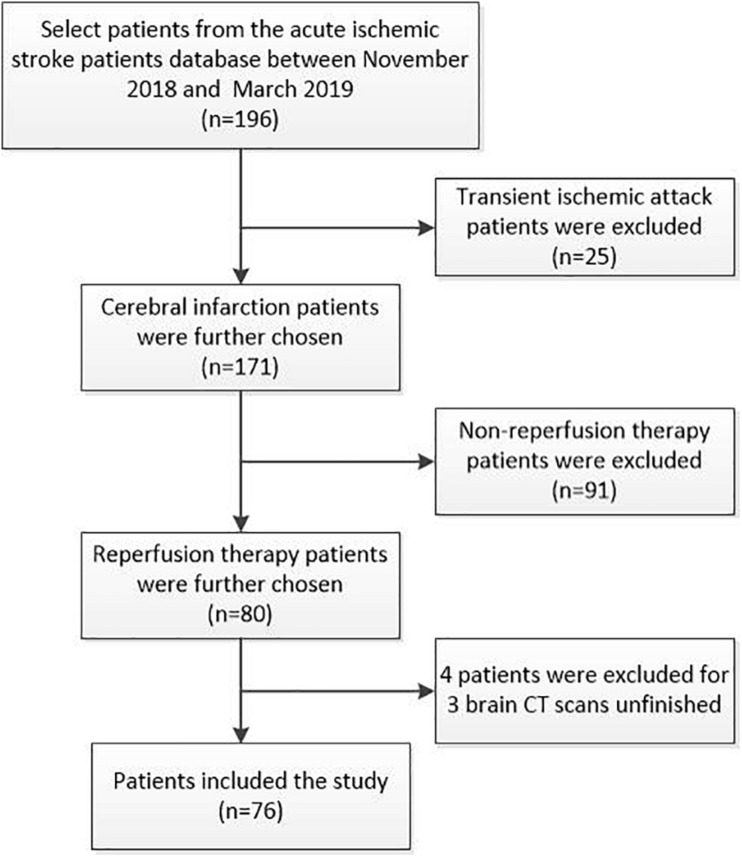
Consort diagram of patient selection process. We screened 196 acute ischemic stroke patients (cerebral infarction: *n* = 171; transient ischemic attack: *n* = 25) from the database between November 2018 and March 2019. In this study, we focused on the stroke patients with reperfusion therapy. According to inclusion and exclusion criteria of the study, transient ischemic attack patients (*n* = 25) and non-reperfusion therapy patients (*n* = 91) were excluded. Finally, 76 cases were included in this study as four patients were excluded due to unfinished brain CT scans within 1 week at admission.

Inclusion criteria: (1) acute ischemic stroke patients with intravenous thrombolysis, mechanical thrombectomy, or bridging therapy (intravenous therapy followed arteries therapy); (2) the time from symptom onset to arriving at the emergency department < 24 h; (3) completed at least three times of brain CT scans within 1 week after reperfusion treatment. Exclusion criteria: (1) no baseline blood samples; (2) inflammatory or infectious diseases, cancer, hemorrhagic disease, and severe renal and liver failure. Informed consents were obtained for data analysis. According to inclusion and exclusion criteria of the study, among the 196 acute ischemic stroke patients in the database, 76 cases were included in this study after excluding patients with transient ischemic attack (*n* = 25), no reperfusion therapy (*n* = 91), and incomplete brain CT scans within 1 week (*n* = 4; Figure 1).

### Baseline Clinical Data

Baseline clinical information for the enrolled patients already existed in our database, including age, sex, medical history, antithrombotic therapy before or after reperfusion, time from onset to blood sampling or reperfusion, baseline blood pressure, baseline NIHSS score, baseline serum occludin level, laboratory data, cranial CT scans, treatment methods, lesion location, and the site of artery occlusion.

Baseline NIHSS score, recorded at admission, was evaluated according to our previous study (Li et al., 2020). Briefly, two neurologists assessed the stroke patients in an emergency department independently. If they had divergence on NIHSS score assessment, a senior doctor would be required to confirm NIHSS score.

Baseline occludin level in serum (blood samples were collected before treatment) was obtained according to our previous study (Li et al., 2020), which was determined using commercial Occludin ELISA Assay Kit (USCN, China).

### Reperfusion Therapies

In this study, the enrolled patients had undergone different reperfusion treatments for acute cerebral infarction. Depending on the specific condition of each patient, a variety of reperfusion procedures were used, including intravenous thrombolysis, mechanical thrombectomy, and bridging therapy (arteries thrombolysis, mechanical thrombectomy, angioplasty with or without stent implantation following intravenous thrombolysis).

### Diagnostic Criteria of HT

Diagnostic criteria for HT in this study followed the guideline of the American Heart Association/American Stroke Association. Briefly, HT was diagnosed in one of the following cases: (1) no intracranial hemorrhage on the first brain CT/MRI scan after cerebral infarction, but occurred on the second cranial CT/MRI examination (Álvarez-Sabín et al., 2013); (2) existing intracranial hemorrhage on the first cranial CT/MRI, calling it a hemorrhagic infarction (Chen et al., 2016). The type of HT was defined according to the ECASS II criteria (Hacke et al., 1998). Hemorrhagic infarction was defined as petechial infarction without a space-occupying effect (HI-1: small petechiae along the edges of the infarction; HI-2 more confluent petechiae in the infarction area without a space-occupying effect). Parenchymal hematoma (PH) was defined as hemorrhage with mass effects, and being associated with symptomatic intracranial hemorrhage with an NIHSS score increase ≥ 4 (Berger et al., 2001) (PH-1: blood clots ≤ 30% of the infarction area with a slight space-occupying effect; PH-2: blood clots in over 30% of the infarction area with an obvious space-occupying effect or clot remote to the infarcted area).

According to the European Cooperative Acute Stroke Study (ECASS) II, most HTs occur within 7 days post stroke onset or recanalization and are the major cause of death (Hacke et al., 1998). Therefore, the endpoint of observation of HT complication was set within 1 week after reperfusion treatment. In this study, all enrolled patients received three times of CT scans to diagnose HT: the first brain CT and the second brain CT/MRI scan was completed within 24 h after reperfusion therapy, and the third CT/MRI was completed within 3–5 days following reperfusion treatment or occurrence of symptomatic intracranial hemorrhage.

### Statistical Analysis

Statistical analysis was performed using SPSS 26.0, R software (version 3.5.1) and MedCalc software. Baseline characteristics were compared between patients with and without HT. Categorical variables were presented as frequencies (%), and continuous variables are shown as means ± SD for normal distribution or medians (interquartile ranges, IQRs) for non-normal distribution. Chi-Squared test or Fisher exact tests were used to assess the association between categorical variables, while *t*-tests and Mann–Whitney *U* tests were used for comparing continuous variables. One-way analysis of variance, followed by LSD-*t* test, was used to examine the difference in each variable among the groups. The significance level of all the statistical tests was set at a type I error rate of 0.05.

We utilized the univariate and multivariate logistic regression analyses to examine the associations of HT with the clinical risk factors, including baseline occludin level and NIHSS score. The variables with *p* < 0.1 from the univariate analyses were included in the multivariate logistic regression model. The variance inflation factor was estimated to check the presence of multicollinearity among variables included in regression model. Receiver operating characteristic (ROC) curve analysis was used to evaluate the prediction performance of individual variables and the model with multiple variables. The unbiased estimate for model prediction accuracy was obtained using leave-one-out cross validation. Area under the curve (AUC) of ROC curves were compared using De-long’s test. The goodness-of-fit of the model was examined using the Hosmer–Lemeshow C statistic.

## Results

### Patient Characteristics

In total, 76 patients were included in the study according to the inclusion and exclusion criteria. Table 1 showed the main characteristics of enrolled patients. Among them, HT occurred in 17 patients (22.37%) within 1 week after reperfusion therapy. Compared with non-HT patients, HT patients had higher median baseline NIHSS score [12 (10.5–18.0) versus 6 (4–12), *p* = 0.003], and higher mean serum occludin level (5.47 ± 1.25 versus 3.81 ± 1.19, *p* < 0.001). There was no significant difference in other vital characteristics between HT and non-HT groups.

**TABLE 1:** Main baseline characteristics of the patients included the group according to the presence or absence of hemorrhagic transformation.

Baseline characteristics	Total patients (*n* = 76)	Non-HT (*n* = 59)	HT (*n* = 17)	*P* value
Male sex, *n* (%)	57 (75.0)	42 (71.2)	15 (88.2)	0.266
Age (years), mean ± SD	64.51 ± 12.90	63.27 ± 12.82	68.82 ± 12.58	0.118
**Medical history, *n* (%)**				
Hypertension	48 (63.2%)	34 (57.6%)	14 (82.4%)	0.115
Diabetes mellitus	30 (39.5%)	25 (42.4%)	5 (29.4%)	0.335
Dyslipidemia	46 (60.5%)	38 (64.4%)	8 (47.1%)	0.197
Coronary heart disease	20 (26.3%)	13 (22.0%)	7 (41.2%)	0.114
Atrial fibrillation	20 (26.3%)	16 (27.1%)	4 (23.5%)	1.000
Current smoking	42 (55.3%)	32 (54.2%)	10 (58.8%)	0.738
Antithrombotic therapy before reperfusion, *n* (%)				0.358
Unused	58 (76.3%)	46 (78.0%)	12 (70.6%)	
Antiplatelet drug	10 (13.2%)	6 (10.2%)	4 (23.5%)	
Anticoagulant drug	8 (10.5%)	7 (11.9%)	1 (5.9%)	
**Clinical measures**				
Time from onset to blood sampling (min), median (IQR)	219.5 (120-300)	210 (120-280)	240 (125-535)	0.214
Time from onset to reperfusion (min), median (IQR)	250 (135-323.75)	250 (135-298)	268 (142-548)	0.254
Baseline NIHSS score, median (IQR)	9 (4.25-13.0)	6 (4-12)	12 (10.5-18.0)	**0.003**
Baseline mRS score, median (IQR)	3.5 (2-4)	3 (2-4)	4 (3-4)	0.175
Baseline SBP (mmHg), median (IQR)	149.5 (130.0-165.8)	149 (129.0-169.0)	153 (143.5-165.0)	0.366
Baseline DBP (mmHg), median (IQR)	80 (71.0-91.0)	78 (70.0-91.0)	86 (76.0-92.0)	0.091
**Location of lesions, *n* (%)**				1.000
Anterior circulation	61 (80.3%)	47 (79.7%)	14 (82.4%)	
Posterior circulation	15 (19.7%)	12 (20.5%)	3 (17.6%)	
**Site of artery occlusion, *n* (%)**				0.610
Middle cerebral artery	42 (55.3%)	31 (52.5%)	11 (64.7%)	
Anterior cerebral artery	1 (1.3%)	1 (1.7%)	0 (0%)	
Internal artery	18 (23.7%)	15 (25.4%)	3 (17.6%)	
Posterior artery	7 (9.2%)	6 (10.2)	1 (5.9%)	
Basilar artery	4 (5.3%)	2 (3.4%)	2 (11.8%)	
Vertebral artery	4 (5.3%)	4 (6.8%)	0 (0%)	
**Treatment methods, *n* (%)**				0.096
Intravenous thrombosis	35 (46.1%)	31 (52.5%)	4 (23.5%)	
Mechanical thrombectomy	22 (28.9%)	15 (25.4%)	7 (41.2%)	
Bridging therapy	19 (25.0%)	13 (22.0%)	6 (35.3%)	
**Antithrombotic therapy after reperfusion, *n* (%)**				0.198
Unused	5 (6.6%)	2 (3.4%)	3 (17.6%)	
Single antiplatelet drug	5 (6.6%)	3 (5.1%)	2 (11.8%)	
Dual antiplatelet drug	47 (61.8%)	40 (67.8%)	7 (41.2%)	
Anticoagulant drug	5 (6.6%)	4 (6.8%)	1 (5.9%)	
Dual antiplatelet drug combined anticoagulant drug	14 (18.4%)	10 (16.9%)	4 (23.5%)	
**Laboratory finding at admission, mean ± SD**				
White blood cell (×10^9^/L)	7.83 ± 2.37	7.80 ± 2.10	7.94 ± 3.25	0.834
Glucose (mmol/L)	8.40 ± 3.38	8.46 ± 3.50	8.21 ± 3.02	0.787
Platelets (×10^9^/L)	205.24 ± 56.00	209.56 ± 55.92	190.24 ± 55.25	0.212
Fibrinogen (g/L)	3.48 ± 1.03	3.54 ± 1.11	3.27 ± 0.72	0.360
Occludin (ng/ml)	4.18 ± 1.40	3.81 ± 1.19	5.47 ± 1.25	** < 0.001**

*HT, hemorrhagic transformation; SD, standard deviation; IQR, interquartile range; NIHSS, National Institutes of Health Stroke Scale; mRS, modified Rankin Scale; SBP, systolic blood pressure; DBP, diastolic blood pressure.*

### Logistic Regression Analysis for the Risk of HT in Acute Ischemic Stroke Patients With Reperfusion Therapies

**FIGURE 2:**
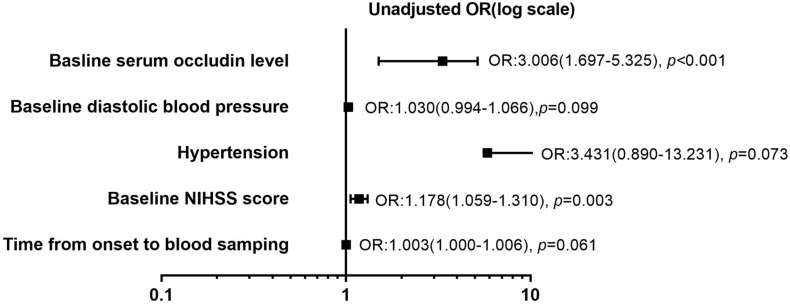
Forest plot of risk factors (*p* < 0.1) for hemorrhagic transformation (HT) in stroke patients with reperfusion therapy in univariate logistic regression analysis. Five potential risk factors (time from onset to blood sampling, baseline diastolic blood pressure, hypertension, baseline National Institute of Health stroke scale (NIHSS) score, and baseline serum occludin level) were analyzed using univariate regression analysis. Forest plot showed that baseline NIHSS score (OR: 1.178, *p* = 0.003) and serum occludin level (OR: 3.006, *p* < 0.001) were the risk factors for HT, respectively. However, the other three parameters (time from onset to blood sampling, baseline diastolic blood pressure, hypertension) were not significantly associated with HT (*p* > 0.05). OR, odds ratio; NIHSS, National Institutes of Health Stroke Scale.

The relationships of clinical variables with the HT in the stroke patients with reperfusion therapy were analyzed using the univariate and multivariate logistic analyses (Table 2). Given the collinearity of time from onset to blood sampling and time from onset to reperfusion (variance inflation factors was significantly >10), the former was selected from the univariate regression. Five variables with *p* < 0.1 from the univariate regression were included in the multivariate analysis. They are the time from onset to blood sampling, baseline diastolic blood pressure, hypertension, baseline NIHSS score, and baseline serum occludin level. Among the five risk factors, baseline serum occludin level and baseline NIHSS score were significantly associated with HT, as the confidence intervals of their odds ratio (OR) do not include one (Figure 2). The variance inflation factors for the five variables were estimated using linear regression and they were all close to one, suggesting that there was no multicollinearity issue in logistic regression with them. The multivariate logistic regression indicated that the baseline serum occludin level (OR 4.969; 95% CI 2.609–11.935, *p* < 0.001) and baseline NIHSS score (OR 1.293; 95% CI 1.079–1.550, *p* = 0.005) were significantly associated with HT even after adjusting the effects of the other three factors (Table 2).

**TABLE 2:** Univariate and multivariate logistic regression analysis to identify factors associated with HT in stroke patients with reperfusion therapy.

Variables	Univariate logistic regression		*p*	Multivariate logistic regression		*p*
	OR	95% CI		OR	95% CI	
Age	1.035	0.991–1.081	0.122	–	–	–
gender	0.329	0.068–1.598	0.168	–	–	–
**Medical history**						
Hypertension	3.431	0.890–13.231	**0.073**	1.278	0.216–7.576	0.787
Diabetes mellitus	0.567	0.177–1.815	0.339	–	–	–
Dyslipidemia	0.491	0.165–1.463	0.202	–	–	–
Coronary heart disease	2.477	0.788–7.787	0.121	–	–	–
Atrial fibrillation	0.827	0.235–2.913	0.767	–	–	–
Current smoking	1.205	0.404–3.597	0.738	–	–	–
Antithrombotic therapy before reperfusion			0.333	–	–	–
Unused	Reference					
Antiplatelet drug	2.556	0.620–10.527	0.194			
Anticoagulant drug	0.548	0.061–4.891	0.590			
**Clinical measures**						
Time from onset to blood sampling	1.003	1.000–1.006	**0.061**	1.005	0.999–1.010	0.081
Time from onset to reperfusion	1.003	1.000–1.006	0.067*	–	–	–
Baseline NIHSS score	1.178	1.059–1.310	**0.003**	1.293	1.079–1.550	**0.005**
Baseline mRS score	1.401	0.788–2.489	0.251	–	–	–
Baseline SBP	1.009	0.990–1.028	0.349	–	–	–
Baseline DBP	1.030	0.994–1.066	**0.099**	1.013	0.958–1.071	0.652
**Location of lesions^#^**	0.839	0.207–3.399	0.806	–	–	–
**Site of arterial occlusion**			0.807	–	–	–
Middle cerebral artery	Reference					
Anterior cerebral artery	0.000		1.000			
Internal artery	0.564	0.137–2.326	0.428			
Posterior cerebral artery	0.470	0.051–4.350	0.506			
Basilar artery	2.818	0.353–22.494	0.328			
Vertebral artery	0.000		0.999			
**Treatment methods**			0.125	–	–	–
Intravenous thrombosis	Reference					
Mechanical thrombectomy	3.617	0.915–14.297	0.067			
Bridging therapy	3.577	0.863–14.817	0.079			
**Antithrombotic therapy after reperfusion**			0.210	–	–	–
Unused	Reference					
Single antiplatelet drug	0.444	0.035–5.581	0.530			
Dual antiplatelet drug	0.117	0.016–0.829	0.032			
Anticoagulant drug	0.167	0.010–2.821	0.214			
Dual antiplatelet drug combined anticoagulant drug	0.267	0.032–2.249	0.224			
**Laboratory examination at admission**						
Glucose	0.977	0.828–1.153	0.783	–	–	–
White blood cell	1.025	0.819–1.283	0.831	–	–	–
Platelets	0.994	0.984–1.004	0.212	–	–	–
Fibrinogen	0.734	0.381–1.416	0.357	–	–	–
Occludin	3.006	1.697–5.325	** < 0.001**	4.969	2.609–11.935	**<0.001**

HT, hemorrhagic transformation; NIHSS, National Institutes of Health Stroke Scale; mRS, modified Rankin Scale; OR, odds ratio; 95% CI, 95% confidence interval.*The variables of time from onset to reperfusion and time from onset to blood sampling have collinearity (Tolerance = 0.001, Variance inflation factor = 1150.978).^#^Location of lesions include two variables of anterior circulation and posterior circulation. Bold values represent different categories and significant p values.

### The Baseline of Serum Occludin and NIHSS Score in Different Types of HT

**FIGURE 3:**
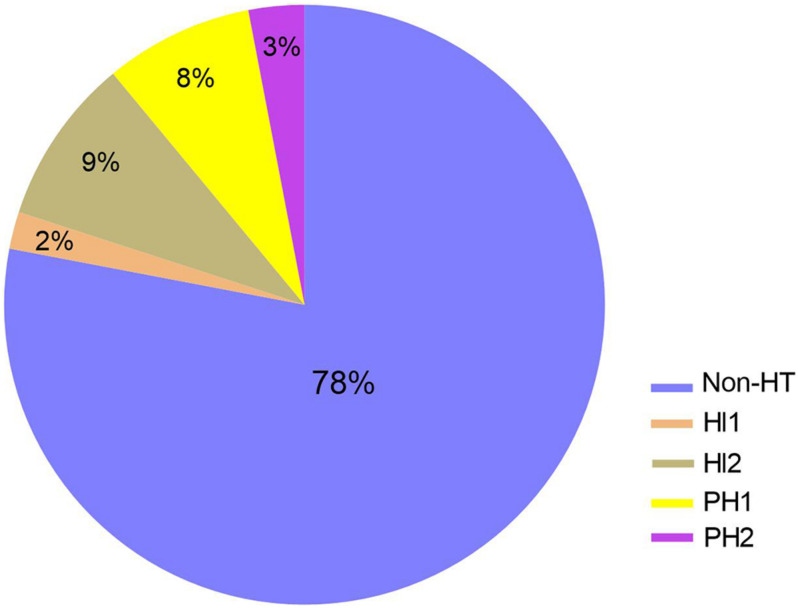
The composition of HT subgroup in stroke patients with reperfusion therapy. The composition of different types of HT was shown in pie graph: HI1 (*n* = 2, 2%), HI2 (*n* = 7, 9%), PH1 (*n* = 6, 8%) and PH2 (*n* = 2, 3%), and Non-HT (*n* = 59, 78%), respectively. HI, hemorrhagic infarction; PH, parenchmal hemtoma.

To further explore the relationship between baseline level of serum occludin and different types of HT, 17 patients with HT were further divided into four subgroups, according to the ECASS II criteria (Hacke et al., 1998): HI1 (*n* = 2), HI2 (*n* = 7), PH1 (*n* = 6), and PH2 (*n* = 2). The composition of different types of HT was shown in pie graph (Figure 3): HI1 (*n* = 2, 2%), HI2 (*n* = 7, 9%), PH1 (*n* = 6, 8%) and PH2 (*n* = 2, 3%), and Non-HT (*n* = 59, 78%), respectively. As there were few cases in HI1 and PH2 groups, HI1 and HI2 was combined as HI, while PH1 and PH2, as PH group for statistical analysis.

**FIGURE 4:**
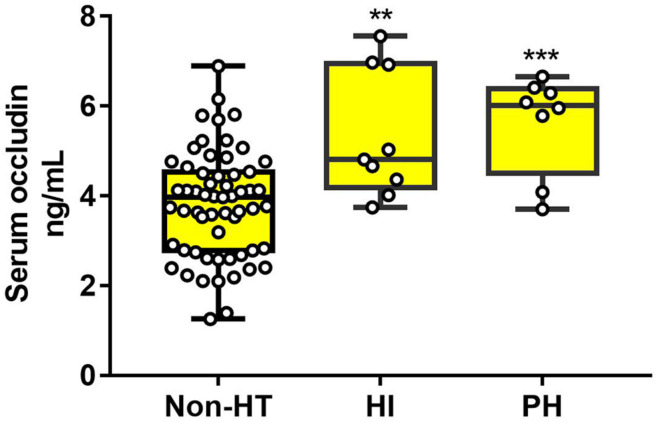
The difference of serum occludin level in HT subgroups and the non-HT group. HT patients (both HI and PH) had a higher serum occludin level than non-HT patients. However, there was no significant difference in serum occludin level between HI and PH. Data were expressed as mean ± SD (***p < 0.001; **p < 0.01; Asterisk indicates PH group and HI group versus non-HT group). HI, hemorrhagic infarction; PH, parenchymal hematoma.

The statistical results showed that HT patients in HI and PH subgroups had much higher levels of serum occludin than non-HT patients (non-HT group:3.81 ± 1.19, HI: 5.34 ± 1.42 and PH:5.62 ± 1.10 ng/ml). But there was no significant difference between HI and PH subgroups (Figure 4). These results suggested that elevated occludin levels were related with the extent of BBB damage, but there is no sufficient evidence for the difference in occludin level between the subtypes of HT.

**FIGURE 5:**
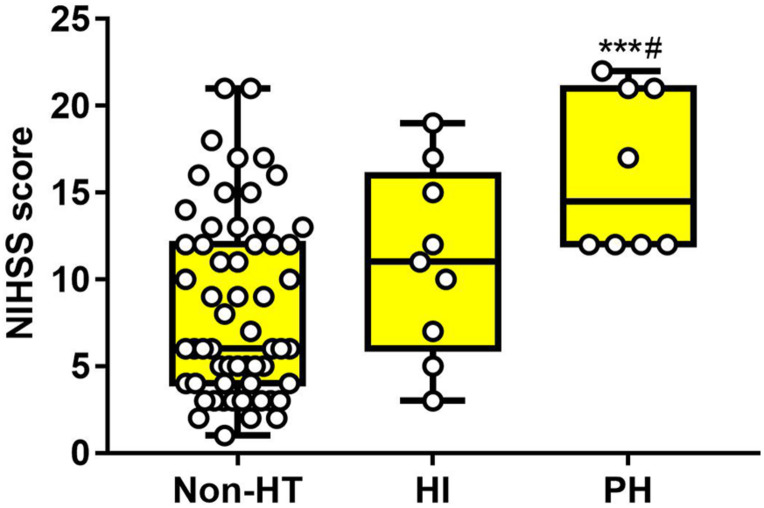
The difference of NIHSS score in HT subgroups and the non-HT group. Baseline NIHSS score of patients with PH was obviously higher than the non-HT group, and slightly higher than the HI group. However, there was no significant difference between non-HT and HI. Data were expressed as mean ± SD (^#^*p* < 0.05; ***p < 0.001, ***PH group versus non-HT group, ^#^PH group versus HI group). HI, hemorrhagic infarction; PH, parenchymal hematoma.

As for baseline NIHSS score, there was significant difference in PH subgroups, compared with the Non-HT group (non-HT: 8.34 ± 5.27, HI: 11.00 ± 5.41, PH: 16.13 ± 4.64). There was no difference between the Non-HT and HI groups (Figure 5). These results suggested that both serum occludin and NIHSS score were related with HT, but neither of them alone was good at predicting the HT or the subgroups of HT.

### Combination of Baseline Serum Occludin and NIHSS Score for Predicting HT in Reperfusion Patients

**FIGURE 6:**
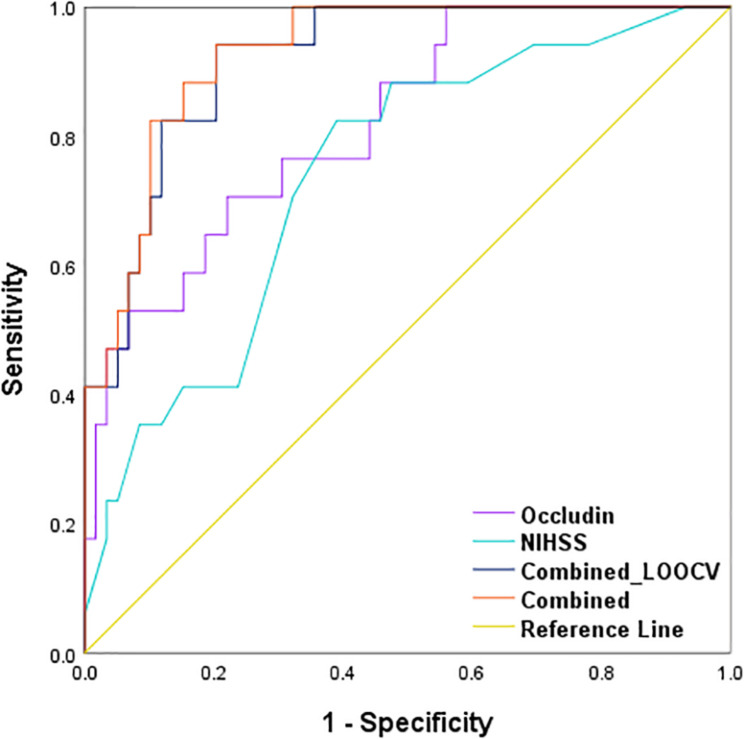
Receiver operating characteristic (ROC) curves of the combination method and single variable to predict HT in stroke patients with reperfusion therapy. AUCs of the combination method (0.928, *p* < 0.001) were higher than serum occludin level (0.821, *p* < 0.001) and baseline NIHSS score (0.740, *p* = 0.003) alone. After using LOOCV, the AUC of the combined method was still higher than the single variable (0.919, *p* < 0.001). These parameters were compared with the Null hypothesis (true area = 0.5). AUC, area under the curve; LOOCV, leave-one-out cross-validation.

Using logistic regression, we developed a combined model with two prediction variables, the baseline serum occludin level and the NIHSS score, to predict the probability of HT event in the stroke patients with reperfusion therapy. The model equation is *logit*(π) = −10.052 + 1.32*x*_1_ + 0.256*x*_2_, where π is the probability of HT, and *x*_1_ and *x*_2_ are the baseline serum occludin level and NIHSS score. The Ngelkerke *R* squared is 0.565, and the *p*-value of the Hosmer-Lemeshow test is 0.755, suggesting the model is a good fit for the data. To obtain an unbiased estimate for the prediction accuracy of the combined model, we performed a leave-one-out cross-validation (LOOCV). The ROC curve based on the LOOCV prediction is presented in Figure 6. The corresponding AUC is 0.919, suggesting that the model has a high prediction accuracy. We also showed the ROC curve of occludin level and that of NIHSS in Figure 6. The AUCs of the two ROC curves are 0.821 and 0.740, which are significantly smaller than that of the combined model (*p*-values of De-long’s test: 0.016 and 0.007) (Table 3). These results indicate that the combined model was more accurate in predicting HT than each individual variable.

**TABLE 3:** The comparison of AUC difference for cross validation curve and risk factors curves.

Variables	AUC difference	95% CI		*p* value
		Lower limit	Upper limit	
Occludin level versus NIHSS score	0.080	–0.107	0.268	0.402
Combined model versus NIHSS score	0.179	0.049	0.309	**0.007**
Combined model versus occludin level	0.099	0.018	0.179	**0.016**

*AUC, area under the curve; NIHSS, National Institutes of Health Stroke Scale; 95% CI, 95% confidence interval. The AUC for combined model was calculated based on the prediction from the leave-one-out cross-validation (LOOCV) procedure. Bold values represent different categories and significant p values.*

## Discussion

This is the first clinical study to explore the value of serum occludin level combined with baseline NIHSS score for predicting the risk of secondary HT in patients with acute cerebral infarction after perfusion therapy. In this study, we found that serum occludin level and baseline NIHSS score were independent risk factors for HT, respectively, and the combination of the two factors could significantly improve the predictive effect for HT. The present study may provide a better method to evaluate the risk of HT before recanalization therapy, helping selecting patients with lower risk of HT.

Reperfusion therapy is widely accepted as an effective treatment method for acute ischemic stroke patients (Campbell et al., 2019). However, reperfusion therapy increases the risk of HT, which offsets the benefits for certain patients (Yaghi et al., 2015). Studies have reported that patients with an asymptomatic small amount of hemorrhage in the cerebral infarction area (HI1, HI2, and PH1) after reperfusion therapy have a poor prognosis at 3 months (Dzialowski et al., 2007). It was also found that these types of asymptomatic hemorrhage may develop into symptomatic hemorrhage (PH2) (Park et al., 2012). Therefore, it is very important to consider patients with asymptomatic HT, as well as symptomatic hemorrhage. In the present study, besides symptomatic HT, we included the patients with an asymptomatic small amount of hemorrhage in the cerebral infarction area (HI1, HI2, and PH1).

Many factors have been suggested to be associated with HT in clinic, such as age, stroke severity, infarct size, hyperglycemia, and hypertension (Whiteley et al., 2012). In this study, we found that the indicator of BBB damage (serum occludin) and stroke severity (baseline NIHSS score) were independent risk factors of HT using multivariate regression analysis.

BBB disruption is a critical step in the development of HT, and therefore, it is useful to evaluate the permeability of BBB as early as possible for predicting the risk of HT (Emberson et al., 2014). Our previous animal study reported that blood occludin level was well-correlated with the extent of BBB damage after ischemic stroke (Pan et al., 2017), and thus serum occludin may serve as a relevant biomarker for assessing the risk of HT before reperfusion therapy (Li et al., 2018). Further clinical study reported that serum occludin level in acute ischemic stroke patients was significantly higher than that in the healthy control group (Shi et al., 2017). In the present clinical study of patients with acute ischemic stroke, serum occludin levels were significantly higher in the HT group (both HI and PH subgroups) than the non-HT group, suggesting that elevated occludin levels were related with the extent of BBB damage, and that serum occludin could predict the risk of secondary HT following reperfusion treatment (Li et al., 2020). However, the AUC of ROC in baseline serum occludin alone was 0.821, which, while it is a solid number, is not high enough for reliably consistently predicting HT. We hypothesized that serum occludin level combined with another clinic index could improve diagnosis effect.

As one of clinic related factors, NIHSS score is considered as a good and stable predictor of bleeding transformation (Emberson et al., 2014). At present, NIHSS score has been brought into many clinic prediction models for HT (Lou et al., 2008; Mazya et al., 2012; Menon et al., 2012; Strbian et al., 2012; Mazya et al., 2013). The present study showed that NIHSS score was significantly higher in PH subgroups, compared with the Non-HT group. However, there was no difference between Non-HT and HI group. This result also suggested that NIHSS score alone needed to be combined with other factors for predicting HT. Multivariate logistic regression showed that baseline NIHSS score was related to HT, and the AUC of NIHSS score reached 0.740, which was consistent with a reported study (Moon et al., 2015). Again, the AUC number is solid but not good enough as a reliable predictor by itself alone. Therefore, it is possible to combine serum occludin with NIHSS score for improving predictive value of HT.

In this study, ROC curve analysis showed that the combination of serum occludin and NIHSS score significantly improved predictive value of HT. In this analysis, we obtained an unbiased estimate for ROC and AUC of the combined model through the LOOCV procedure. De-long’s test results showed that, at 0.919, the AUC of the combined model is significantly larger than that of an individual predictor, suggesting that a combined diagnosis method is superior to a single variable (occludin level or NIHSS score) alone. Therefore, the combination of serum occludin levels (representing the extent of BBB disruption) and NIHSS score (reflecting stroke severity) markedly increased the predictive value for HT, providing a new method for predicting HT in stroke patients with reperfusion therapy.

### Limitations of the Study

Although we have demonstrated that the combined diagnosis method for HT was better than each individual index in this study, the small sample size could bring about some limitations. These results, including risk factors for HT and the AUC of ROC curves obtained from the database may have false positives or biases. Therefore, these results need to be further confirmed in a prospective study with a larger number of patients. Moreover, a larger number of cases may include more HT cases, which would be more helpful to analyze the subgroup of symptomatic HT and provide a more useful guideline for clinical practice.

## Conclusion

Baseline serum occludin level and baseline NIHSS score were independent risk factors for secondary HT in patients with acute cerebral infarction following perfusion therapy. The combination of the two risk factors may provide an early prediction for HT in patients with acute cerebral infarction with reperfusion treatment.

## Data Availability Statement

The original contributions presented in the study are included in the article/supplementary material, further inquiries can be directed to the corresponding authors.

## Ethics Statement

The studies involving human participants were reviewed and approved by Ethics Committee of Xuanwu Hospital, Capital Medical University. The ethics committee waived the requirement of written informed consent for participation.

## Author Contributions

SY: analysis of the data, design of the study, and drafting the manuscript for intellectual content. WL, CH, and HK: analysis of the data and revising the manuscript. QM and XJ: analysis of the data and interpretion of the data. ZQ and KL: design of the study, analysis of the data, and revising the manuscript. All authors contributed to the article and approved the submitted version.

## Conflict of Interest

The authors declare that the research was conducted in the absence of any commercial or financial relationships that could be construed as a potential conflict of interest.

## Publisher’s Note

All claims expressed in this article are solely those of the authors and do not necessarily represent those of their affiliated organizations, or those of the publisher, the editors and the reviewers. Any product that may be evaluated in this article, or claim that may be made by its manufacturer, is not guaranteed or endorsed by the publisher.

## References

[B1] Álvarez-Sabín J. Maisterra O. Santamarina E. Kase C. S. (2013). Factors influencing haemorrhagic transformation in ischaemic stroke. *Lancet Neurol.* 12 689–705. 10.1016/s1474-4422(13)70055-3 23726850

[B2] Berger C. Fiorelli M. Steiner T. Schäbitz W. R. Bozzao L. Bluhmki E. (2001). Hemorrhagic transformation of ischemic brain tissue: asymptomatic or symptomatic? *Stroke* 32 1330–1335. 10.1161/01.str.32.6.1330 11387495

[B3] Campbell B. C. V. De Silva D. A. Macleod M. R. Coutts S. B. Schwamm L. H. Davis S. M. (2019). Ischaemic stroke. *Nat. Rev. Dis. Primers* 5:70. 10.1038/s41572-019-0118-8 31601801

[B4] Chen G. Wang A. Zhao X. Wang C. Liu L. Zheng H. (2016). Frequency and risk factors of spontaneous hemorrhagic transformation following ischemic stroke on the initial brain CT or MRI: data from the China National Stroke Registry (CNSR). *Neurol. Res.* 38 538–544. 10.1080/01616412.2016.1187864 27320249

[B5] Dzialowski I. Pexman J. H. Barber P. A. Demchuk A. M. Buchan A. M. Hill M. D. (2007). Asymptomatic hemorrhage after thrombolysis may not be benign: prognosis by hemorrhage type in the Canadian alteplase for stroke effectiveness study registry. *Stroke* 38 75–79. 10.1161/01.str.0000251644.76546.62 17122437

[B6] Emberson J. Lees K. R. Lyden P. Blackwell L. Albers G. Bluhmki E. (2014). Effect of treatment delay, age, and stroke severity on the effects of intravenous thrombolysis with alteplase for acute ischaemic stroke: a meta-analysis of individual patient data from randomised trials. *Lancet* 384 1929–1935. 10.1016/s0140-6736(14)60584-5 25106063 PMC4441266

[B7] Hacke W. Kaste M. Fieschi C. von Kummer R. Davalos A. Meier D. (1998). Randomised double-blind placebo-controlled trial of thrombolytic therapy with intravenous alteplase in acute ischaemic stroke (ECASS II). Second European-Australasian Acute Stroke Study Investigators. *Lancet* 352 1245–1251. 10.1016/s0140-6736(98)08020-9 9788453

[B8] Hawkins B. T. Davis T. P. (2005). The blood-brain barrier/neurovascular unit in health and disease. *Pharmacol. Rev.* 57 173–185. 10.1124/pr.57.2.4 15914466

[B9] Kazmierski R. Michalak S. Wencel-Warot A. Nowinski W. L. (2012). Serum tight-junction proteins predict hemorrhagic transformation in ischemic stroke patients. *Neurology* 79 1677–1685. 10.1212/wnl.0b013e31826e9a83 22993287

[B10] Li W. Pan R. Qi Z. Liu K. J. (2018). Current progress in searching for clinically useful biomarkers of blood-brain barrier damage following cerebral ischemia. *Brain Circ.* 4 145–152. 10.4103/bc.bc_11_18 30693340 PMC6329218

[B11] Li W. Qi Z. Kang H. Qin X. Song H. Sui X. (2020). Serum occludin as a biomarker to predict the severity of acute ischemic stroke, hemorrhagic transformation, and patient prognosis. *Aging Dis.* 11 1395–1406. 10.14336/ad.2020.0119 33269096 PMC7673856

[B12] Lou M. Safdar A. Mehdiratta M. Kumar S. Schlaug G. Caplan L. (2008). The HAT Score: a simple grading scale for predicting hemorrhage after thrombolysis. *Neurology* 71 1417–1423. 10.1212/01.wnl.0000330297.58334.dd 18955684 PMC2676961

[B13] Mazya M. Egido J. A. Ford G. A. Lees K. R. Mikulik R. Toni D. (2012). Predicting the risk of symptomatic intracerebral hemorrhage in ischemic stroke treated with intravenous alteplase: safe Implementation of Treatments in Stroke (SITS) symptomatic intracerebral hemorrhage risk score. *Stroke* 43 1524–1531. 10.1161/strokeaha.111.644815 22442178

[B14] Mazya M. V. Bovi P. Castillo J. Jatuzis D. Kobayashi A. Wahlgren N. (2013). External validation of the SEDAN score for prediction of intracerebral hemorrhage in stroke thrombolysis. *Stroke* 44 1595–1600. 10.1161/strokeaha.113.000794 23632975

[B15] Menon B. K. Saver J. L. Prabhakaran S. Reeves M. Liang L. Olson D. M. (2012). Risk score for intracranial hemorrhage in patients with acute ischemic stroke treated with intravenous tissue-type plasminogen activator. *Stroke* 43 2293–2299. 10.1161/strokeaha.112.660415 22811458

[B16] Moon B. H. Park S. K. Jang D. K. Jang K. S. Kim J. T. Han Y. M. (2015). Use of APACHE II and SAPS II to predict mortality for hemorrhagic and ischemic stroke patients. *J. Clin. Neurosci.* 22 111–115. 10.1016/j.jocn.2014.05.031 25172016

[B17] Pan R. Yu K. Weatherwax T. Zheng H. Liu W. Liu K. J. (2017). Blood occludin level as a potential biomarker for early blood brain barrier damage following ischemic stroke. *Sci. Rep.* 7:40331. 10.1038/srep40331 PMC5228160 28079139

[B18] Park J. H. Ko Y. Kim W. J. Jang M. S. Yang M. H. Han M. K. (2012). Is asymptomatic hemorrhagic transformation really innocuous? *Neurology* 78 421–426. 10.1212/wnl.0b013e318245d22c 22282643

[B19] Shi S. Qi Z. Ma Q. Pan R. Timmins G. S. Zhao Y. (2017). Normobaric hyperoxia reduces blood occludin fragments in rats and patients with acute ischemic stroke. *Stroke* 48 2848–2854. 10.1161/strokeaha.117.017713 28931617 PMC5659343

[B20] Strbian D. Meretoja A. Ahlhelm F. J. Pitkäniemi J. Lyrer P. Kaste M. (2012). Predicting outcome of IV thrombolysis-treated ischemic stroke patients: the DRAGON score. *Neurology* 78 427–432. 10.1212/wnl.0b013e318245d2a9 22311929

[B21] Tan S. Wang D. Liu M. Zhang S. Wu B. Liu B. (2014). Frequency and predictors of spontaneous hemorrhagic transformation in ischemic stroke and its association with prognosis. *J. Neurol.* 261 905–912. 10.1007/s00415-014-7297-8 24590407

[B22] Wang W. Jiang B. Sun H. Ru X. Sun D. Wang L. (2017). Prevalence, incidence, and mortality of stroke in china: results from a nationwide population-based survey of 480 687 adults. *Circulation* 135 759–771. 10.1161/circulationaha.116.025250 28052979

[B23] Whiteley W. N. Slot K. B. Fernandes P. Sandercock P. Wardlaw J. (2012). Risk factors for intracranial hemorrhage in acute ischemic stroke patients treated with recombinant tissue plasminogen activator: a systematic review and meta-analysis of 55 studies. *Stroke* 43 2904–2909. 10.1161/strokeaha.112.665331 22996959

[B24] Yaghi S. Boehme A. K. Dibu J. Leon Guerrero C. R. Ali S. Martin-Schild S. (2015). Treatment and outcome of thrombolysis-related hemorrhage: a multicenter retrospective study. *JAMA Neurol.* 72 1451–1457. 10.1001/jamaneurol.2015.2371 26501741 PMC4845894

